# Freshwater Plants Synthesize Sulfated Polysaccharides: Heterogalactans from Water Hyacinth (*Eicchornia crassipes*)

**DOI:** 10.3390/ijms13010961

**Published:** 2012-01-17

**Authors:** Nednaldo Dantas-Santos, Dayanne Lopes Gomes, Leandro Silva Costa, Sara Lima Cordeiro, Mariana Santos Santana Pereira Costa, Edvaldo Silva Trindade, Célia Regina Chavichiolo Franco, Kátia Castanho Scortecci, Edda Lisboa Leite, Hugo Alexandre Oliveira Rocha

**Affiliations:** 1 Laboratory of Biotechnology of Natural Polymers (BIOPOL), Department of Biochemistry, Federal University of Rio Grande do Norte (UFRN), Natal-RN 59078-970, Brazil; E-Mails: nednaldod@hotmail.com (N.D.-S.); dayanne_gomes@hotmail.com (D.L.G.); leandro-silva-costa@hotmail.com (L.S.C); sara-cordeiro@hotmail.com (S.L.C.); marispc_bio@yahoo.com.br (M.S.S.P.C.); 2 Health Post-Graduate Program, Federal University of Rio Grande do Norte (UFRN), Natal-RN 59078-970, Brazil; 3 Department of Cell Biology, Federal University of Parana (UFPR), Curitiba-PR 81531-990, Brazil; E-Mails: estrindade@ufpr.br (E.S.T.); crcfranc@ufpr.br (C.R.C.F); 4 Department of Cell Biology and Genetic, Federal University of Rio Grande do Norte (UFRN), Natal-RN 59078-970, Brazil; E-Mail: kacscort@yahoo.com; 5 Laboratory of Glycobiology, Department of Biochemistry, Federal University of Rio Grande do Norte (UFRN), Natal-RN 59078-970, Brazil; E-Mail: eddaleite@cb.ufrn.br

**Keywords:** freshwater, biological activities, sulfated galactan

## Abstract

Sulfated polysaccharides (SP) are found mainly in seaweeds and animals. To date, they have only been found in six plants and all inhabit saline environments. Furthermore, there are no reports of SP in freshwater or terrestrial plants. As such, this study investigated the presence of SP in freshwaters *Eichhornia crassipes*, *Egeria densa*, *Egeria naja*, *Cabomba caroliniana*, *Hydrocotyle bonariensis* and *Nymphaea ampla*. Chemical analysis identified sulfate in *N. ampla*, *H. bonariensis* and, more specifically, *E. crassipes*. In addition, chemical analysis, FT-IR spectroscopy, histological analysis, scanning electron microscopy (SEM) and energy-dispersive X-ray analysis (EDXA), as well as agarose gel electrophoresis detected SP in all parts of *E. crassipes*, primarily in the root (epidermis and vascular bundle). Galactose, glucose and arabinose are the main monosaccharides found in the sulfated polysaccharides from *E. crassipes*. In activated partial thromboplastin time (APTT) test, to evaluate the intrinsic coagulation pathway, SP from the root and rhizome prolonged the coagulation time to double the baseline value, with 0.1 mg/mL and 0.15 mg/mL, respectively. However, SP from the leaf and petiole showed no anticoagulant activity. *Eichornia* SP demonstrated promising anticoagulant potential and have been selected for further studies on bioguided fractionation; isolation and characterization of pure polysaccharides from this species. Additionally *in vivo* experiments are needed and are already underway.

## 1. Introduction

Sulfated polysaccharides (SP) from different sources have been studied in the light of their important pharmacological activities, such as anticoagulant, antioxidant, antiproliferative, antitumoral, anticomplementary, anti-inflammatory, and antiviral properties [1]. Anticoagulant activity is among the most widely studied properties of sulfated polysaccharides. Unfractionated and low molecular weight heparins are the only sulfated polysaccharides currently used as anticoagulants. However, these compounds have several side effects including bleeding and thrombocytopenia, increasing the need to search for alternative sources of anticoagulant agents, such as other SP.

SP are found mainly in marine seaweeds [2] and the animal kingdom [3]. Sulfated glycosaminoglycans (GAGs) are the best-known animal SP. They are polydisperse linear polysaccharides composed of alternate units of hexosamine and uronic acid, connected by glycosidic linkages. Sulfation takes place at different positions of the disaccharide units. Almost all GAGs occur in tissues as proteoglycans (PGs), where the polysaccharide chains are covalently linked to a core protein [4]. GAGs display peculiar structural variability according to tissue and species. Considering their cellular localization, structural diversity, and expression changes during different physiological conditions, including cell division, cell growth, cell adhesion, cell migration, cell differentiation, *etc*., then this raises the hypothesis that PGs and GAGs play a specific role in cellular interactions.

In seaweeds, SP are found in the extracellular matrix. Their physiological function in seaweeds remains little known, although some authors suggest they protect against dehydration occurring at low tide. This would explain the higher SP content of seaweeds at inter-tidal level [5]. The SP structures depend on the seaweed classes Rhodophyceae, Phaeophyceae and Chlorophyceae. The most well known SP in Rhodophyceae are carragenans and agarans, which are sulfated galactans [6]. SP from brown Phaeophyceae are homo- and hetero-polysaccharides containing α-l-sulfated fucose called fucan and fucoidan, respectively [7]. SP found in Chlorophyceae are usually heteropolysaccarides containing xylose, galactose, arabinose, mannose, glucuronic acid or glucose. However, there is a predominance of one monosaccharide at the expense of others in several algal orders [8].

Since SP in seaweeds and animals may participate in and regulate many cellular events and physiological processes in several organisms displaying tissue organization [2,3], many researchers believe SP could also occur in other types of organisms with tissue organization. However, there are no studies showing fungi synthesizing SP and only recently Aquino and colleagues confirmed the presence of SP in plants, namely three species of marine angiosperms, *Ruppia maritima*, *Halodule wrightii* and *Halophila decipiens*, and two species of mangrove, *Avicennia schaueriana* and *Rhizophora mangle*. However, the authors did not find SP in terrestrial plants *Zea mays* (corn), *Oryza sativa* (rice) or *Phaseolus vulgaris* (bean); neither did they investigate SP in freshwater plants [9,10].

In the eighties, Nader and colleagues studied 15 species of invertebrate groups including Crustacea, Pelecypoda and Gastropoda, and recorded a positive correlation between sulfated polysaccharide concentrations and water salinity in these aquatic invertebrates [11]. In addition, SP synthesized in marine angiosperm *Ruppia maritima* was not found when the plant was cultivated in fresh water [10]. Thus, the question, do freshwater plants actually synthesize SP?

In order to understand this question, the present study used different tools such as chemical and histological analyses, energy-dispersive X-ray analysis (EDXA), gel electrophoresis and infra-red spectroscopy to confirm the presence of sulfated polysaccharides in freshwater plants for the first time. Moreover, we also demonstrate that SP extracted from *E. crassipes* root has potential as an anticoagulant compound.

## 2. Results and Discussion

### 2.1. Identification of Freshwater Plants That Synthesize Sulfated Polysaccharides

Abiotic factors can affect the physical and chemical characteristics of river water, which can, in turn, affect molecular synthesis in plants. Salinity has been suggested as a factor that may induce SP production in animals and seaweeds [10,11]. As such, we selected a river containing several species of freshwater plants and low or no salinity as our collection point, namely the Agua Quente stream. Several abiotic parameters were analyzed. Average rainfall was 252.0 mm/month and sun exposure was 248.20 h per month. During analysis, average water temperature in the stream remained around 32 °C. We also recorded an absence of salinity and no changes in water depth during the sampling period.

Seven freshwater plants were collected in the Agua Quente stream: *Eichhornia crassipes*, *Egeria densa*, *Egeria cobra*, *Cabomba caroliniana*, *Ceratophyllum aquaticum*, *Hydrocotyle bonariensis* and *Nymphaea ample*. These were collected and divided into root, rhizome, petiole, leaf, and flower, when possible. Each portion was submitted separately for polysaccharide extraction as described in Methods. Polysaccharides; from each plant, portions were obtained after proteolysis and precipitation with methanol. Chemical analysis showed the presence of sulfate and polysaccharides in three from the seven plants analyzed as presented at Table 1. No preference was found for sulfate specific location in plant tissue. *N. ampla* showed higher amounts of sulfate in the root, *H. bonariensis* in leaves and *E. crassipes* in petiole and root. However, we still detected levels of proteins in our preparation. In order to rule out the possibility that the sulfate in some samples could be derived from the proteins, but not polysaccharides, the proteins in the samples were precipitated with trichloroacetic acid (TCA) (80%). Then, the amount of sulfate and proteins in the samples was re-determined and the data showed that the amount of sulfate did not change after TCA treatment (data not shown). Additionally, we did not detect any protein in all of the samples analyzed.

Chemical data obtained showed that three of the seven plants collected in fresh water contained SP (*E. crassipes*, *H. bonariensis* and *N. ampla*). This indicates SP is more common in plants than previously thought. Since *E. crassipes* exhibited the highest amount of sulfate in comparison to other plants, it was chosen for the next set of experiments.

### 2.2. Characterization of Sulfated Polysaccharides from *E. crassipes*

*E. crassipes*, more commonly known as aguape (Brazil) or water hyacinth (USA), has been studied by other groups, but has never been investigated for the presence of SP. As such, percentage composition was analyzed for comparison with previous proximate composition determination, since it may have been different. Proximate composition of *E. crassipes* portions (leaves, petioles, rhizome and roots) is shown in Table 2. All parts displayed low lipid and nitrogen contents. Moisture values ranged from 86 (leaves) to 93 (petiole and rhizome), with no significant differences (*p* > 0.05) among replicates. No significant differences were recorded when comparing the percentage of carbohydrates in the four plant sections. The highest ash content was found in the root and rhizome, while the largest percentage of protein was observed in the petioles and leaves.

Data obtained for different portions of *E. crassipes* (Table 2) was comparable to that recorded in prior research [12]. This confirms that *E. crassipes* used here is similar to that previously investigated.

Monosaccharide compositions (Table 3) from *E. crassipes* polysaccharide showed that galactose, glucose, arabinose, xylose are present in all parts. In addition, mannose and xylose were also found in the root and rhizome. Galactose was the main monosaccharide in polysaccharide fractions from *E. crassipes*.

No amino sugars were identified in the monosaccharide composition of SP in *E. crassipes*. This result refutes the hypothesis that *E. crassipes* may be capable of synthesizing glycosaminoglycans as occurs in animals. Furthermore, fucose, a sugar type found primarily in brown seaweeds, was not present. Monosaccharide composition also indicated the presence of galactose, as well as small amounts of glucose and arabinose. SP with a similar composition are observed in green seaweed [8], although sulfated homogalactans have also been described [13]. Sulfated homogalactans have been characterized in SP from the seagrass *R. maritima*, *H. decipiens*, *H. wrightii* [9], while those in mangroves were sulfated arabinogalactans. These data demonstrate that SP in *E. crassipes* are more similar to those found in other plants and in green seaweeds than those produced by brown seaweeds and animals.

Characteristic sulfate absorptions were identified in the all FT-IR spectra of *E. crassipes* polysaccharides: bands approximately 1252 cm^−1^ for an asymmetric S=O stretching vibration [14]; bands around 1068–1167 cm^−1^ were assigned mainly to symmetric O=S=O stretching vibration of sulfate esters [14] found in all spectra. Bands around 820 cm^−1^ were recorded in all spectra, indicating that sulfate groups are located at position six of the galactose ring [15,16]. Additionally, at 3000–3400 cm^−1^ and around 2920 cm^−1^ all polysaccharides showed signs of the stretching vibration O–H and C–H, respectively. Bands at about 1638–1654 cm^−1^ were due to bound water [16].

### 2.3. Polysaccharide Analysis by Agarose Gel Electrophoresis

**Figure 1:**
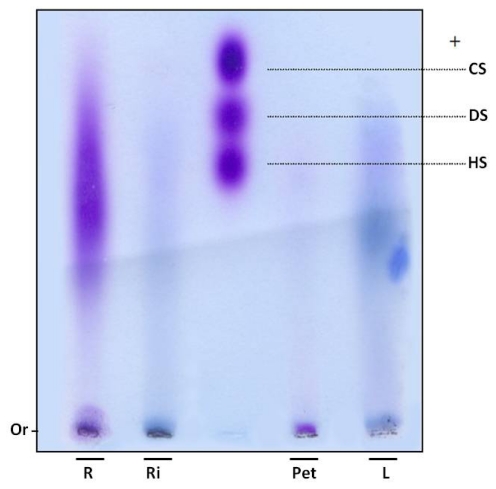
Electrophoresis of sulfated polysaccharides from *E. crassipes*. Aliquots of approximately 5 μL (250 μg) of Eicchonia polysaccharides or (5 μg) glycosaminoglycans were applied in agarose gel (10 × 7.5 cm, and 0.2 cm thick) prepared in 0.05 M 1,3-diaminopropane-acetate buffer pH 9.0, and subjected to electrophoresis at 110 V/cm for 60 min. Gels were then held in 0.1% cetyltrimethylammonium bromide for 2 h and dried. Polysaccharides were stained with 0.1% toluidine blue in a solution containing 50% ethanol and 1% acid acetic, in water, for 15 min. Gels were then de-stained using the same solution, without toluidine blue. R—root; Ri—rizhome; Pet—petiole; L—leaf; CS—chondroitin sulfate; DS—dermatan sulfate; HS—heparan sulfate; Or—origin.

In order to verify whether sulfate ions were linked to polysaccharides, SP were subjected to electrophoresis in agarose gel. Gels were dried and stained with toluidine blue. Using this dye, polysaccharides are stained purple, as with the GAGs heparan, chondroitin and dermatan sulfate (Figure 1). However, purple bands were not observed in the leaves and rhizome fractions of *E. crassipes*. Moreover, the root portion showed polydisperse band, purple staining, indicating the presence of sulfated polysaccharides (Figure 1) and the petiole displayed a small purple band with low electrophoretic mobility, since it did not move from the gel line.

SP were confirmed in the petiole and roots by agarose gel electrophoresis. An interesting aspect is that these two portions also had a high proportion of sulfate in relation to sugar (Table 1). The 1,3-diaminopropane/acetate buffer used in electrophoresis allowed diamines to interact with sulfate groups present in SP, reducing electrophoretic mobility. This interaction also depends on conformation and consequently the composition of SP [17]. Thus chondroitin sulfate and dermatan sulfate, which have the same charge/mass ratio, have different electrophoretic mobilities in a diaminopropane/acetate buffer (Figure 1).

Since SP from the petiole and root portions exhibited different mobility, *E. crassipes* may produce more than one type of SP. This finding was previously recorded in different organisms, such as invertebrates and vertebrates, as well as seaweeds. Purple bands were not detected in the rhizome and leaves. However, infrared analysis indicated these sections also produce SP, albeit probably in small amounts, given it was not detected by agarose gel methodology.

### 2.4. Histological, and EDXA/MEV Analysis

**Figure 2:**
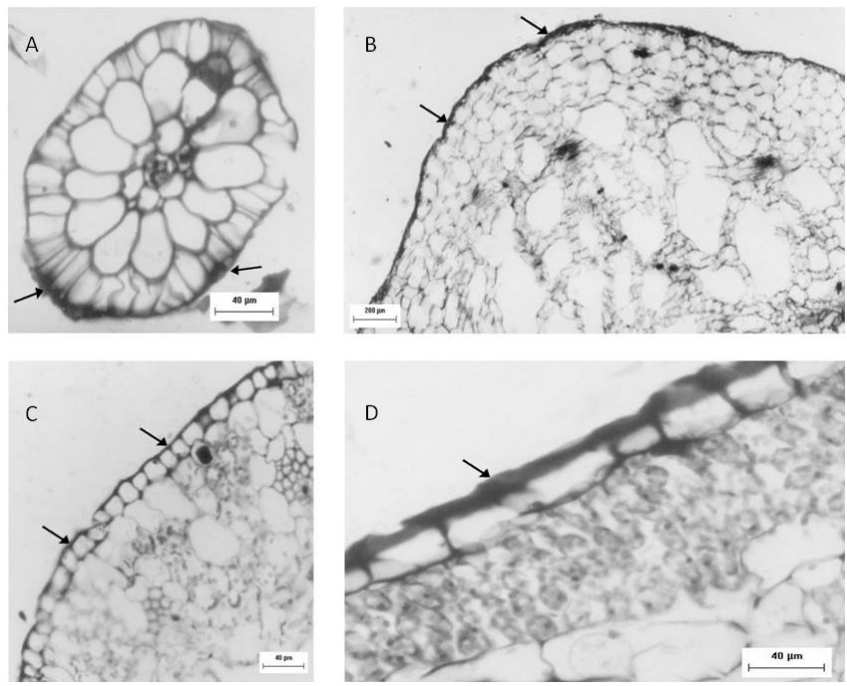
Sulfated polysaccharide localization in different regions of the *E. crassipes* determined by histological analysis. Optical microscopy images of the root (**A**), rhizome (**B**) petiole (**C**) and leaf (**D**) of the *E. crassipes* showed differences in the staining intensity from toluidine blue represented by the arrows.

Another approach used to identify sulfated polysaccharides in this plant was histological localization analysis of each portion. For freshwater *Eicchornia crasipes*, sulfated polysaccharide was found primarily in the cell wall, after these sections were stained with toluidine blue and observed with optical microscopy (Figure 2). Sulfated polysaccharides were detected in the epidermis and exodermis at the cortex of the root (Figure 2A). The rhizome was also observed, exhibiting irregular cells in the cortex (marked with an arrow) and a well organized layer of cells in the epidermis (indicated by an arrow). This section also showed sulfated polysaccharides in the cortical region of the rhizome (marked with an arrow), but displayed less labeling intensity compared with the root (Figure 2B). The third area analyzed for *E. crasipes* was the petiole, which presented an epidermal layer of cells with uniform size similar to those found in the rhizome region. This was followed by collenchymas, composed of cells with different shapes and sizes. The epidermis and collenchymas were stained with toluidine blue, indicating the presence of sulfated compounds in these areas (Figure 2C). The *E. crasipes* leaf is formed of a layer of cuboidal-shaped cells external to the leaf tissue, followed by successive layers of cells of varying sizes and shapes. The color of the epidermal cell layer in the leaf region suggested the presence of sulfated compounds given that it was stained by toluidine blue when compared to other regions (2D). However, as observed with the rhizome, the leaf also demonstrated less labeling intensity.

**Figure 3:**
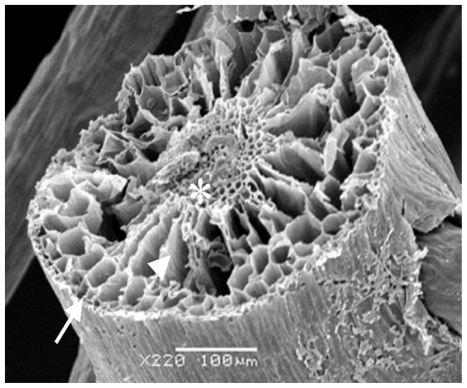
Scanning electron microscopy from *E. crassipes* root. Arrow, arrowhead, and asterisk indicate the points of collection of data for analysis of energy-dispersive X-ray analysis (EDXA). Arrow—epidermis; arrowhead—cortex; asterisk—vascular bundle.

Considering all the results obtained, the rizhome portion displayed the highest amount of SP. Thus, this section was analyzed using scanning electron microscopes/Energy-dispersive X-ray spectroscopy (SEM/EDXA) in order to verify the presence of sulfur from SP. Figure 3 shows three points analyzed with this method. SEM/EDXA analysis of the plant root allowed simultaneous observation of several elements in a fast and quantitative manner (Table 4). Considering the three triplicate analyses, elements C, O and N, as well as smaller amounts of S were detected, confirming the presence of sulfate polysaccharide in *E. crasipes*.

Histochemical analysis identified SP in all parts analyzed. As observed in *R. maritima* [9], substantial amounts of SP were also found in the roots of *E. crassipes.* Aquino and colleagues suggested its presence in the roots may be related to the role of SP in nutrition and water capture. Results obtained by EDXA showed sulfur in the epidermis and cell wall, important regions for water and nutrient capture in plants, reinforcing the function of SP. However, what exactly is the role of SP in the leaves and petiole of *E. crassipes*? It has been suggested that SP in seaweeds may protect against water loss during sun exposure at low tide [5]. As such, SP may have the same function in the leaves and petiole of *E. crassipes*.

Aquino and colleagues cultivated the seaweed *R. maritima* in different tanks under different salinity. SP were produced in smaller amounts in *R. maritima* plants grown in low salinity and in fresh water, no SP were detected. However, in rice (*O. sativa*) cultivated in the presence or absence of 200 mM of NaCl, no SP production was observed in the presence of salinity [10]. Furthermore, our results with *E. crassipes* collected in a non-saline environment confirmed the presence of SP. This suggests freshwater plants may produce SP that is not related to salinity stress, and that other factors may promote their production.

### 2.5. Genomic Analysis

Considering this hypothesis and genomic tools available on web pages, an *in silico* analysis was conducted to identify possible sequences related to SP synthesis in plants. Since these sequences were not found in the genome database for plants and seaweeds, sequences were used from *Rattus norvegicus* (M92042); *Homo sapiens* (U17970) and *Mus musculus* (U02304) to design two pairs of primers at the region corresponding to 5′PSB 3′PB—that recognizes 3′-phosphoadenosine-5′-phosphosulfate (PAPS) binding motif. First, a polymerase chain reaction (PCR) amplification was performed using rat and human DNA to test the primers. Products obtained were sequenced and corresponded to these two sequences. Next, PCR amplification was carried out using *E. crassipes* DNA in high and low stringent PCR conditions. The resulting products obtained were cloned and sequenced. However, no sequences related to sulfotransferase were obtained. The outcome indicated these regions may be divergent in plants, given that the sequences could not be amplified.

### 2.6. Anticoagulant Activity

Several investigations have shown that SP have different biological/pharmacological activities, such as anticoagulants [8,18]. The present study demonstrates that *E. crassipes* produces SP, making it important to evaluate the polysaccharide bioactivity of this freshwater plant. Thus, we assessed anticoagulant activity of sulfated polysaccharide-rich extracts of *E. crassipes* through activated partial thromboplastin time (APTT) and protrombin time (PT) tests, which analyze the intrinsic and extrinsic pathways of coagulation, respectively. Since this is the first identification of SP in freshwater plants, we also examined anticoagulant activity in three seaweeds: *Caulerpa sertularioides* (green), *Gracilaria caudata* (red), and *Dictyopteris delicatula* (brown). These are known to have anticoagulation activity and as such were used for comparison with *E. crassipes* SP.

The anticoagulant activity is presented in Table 5. In the PT test, SP produced by *E. crassipes* could not alter the plasma clotting time. However, in APTT testing, several polysaccharides showed considerable anticoagulant activity. SP from *D. delicatula* was the most potent anticoagulant compound, followed by SP in the roots and leaves of *E. crassipes*. (Table 5). No correlation was found between anticoagulant activity and sulphate/sugar ratio (*R*^2^ = 0.201). This is in agreement with different results showing that the anticoagulant effect of SP is dependent on molecule structure and charge distribution, not only on the sulfate group [19,20]. None of the sulfated polysaccharides assayed were more active than heparin, an anticoagulant drug used as the positive control.

In addition to the content of sulfate groups in polysaccharides, the positions of these groups in a monosaccharide residue also influenced the anticoagulant activity [21]. Here, SP from *E. crassipes* contained galactose-6-sulfate as observed in FT-IR, indicating this sulfated residue to be important for anticoagulant activity of this SP. In agreement with our results, Chaidedgumjorn *et al*. [22] showed that polysaccharides specifically desulfated at position C6 lost their anticoagulant activity, whereas desulfation on C4 did not affect polysaccharide anticoagulant activity.

*E. crassipes* is a very resilient plant that adapts well to many aquatic environments, including those affected by human activities. It is one of the most proliferate plant types and is easily cultivated, already colonizing 62 countries in Africa, Asia and North America, with human help. In some areas, *E. crassipes* is used in phytoremediation projects. However, in most cases it is considered a pest [23,24]. Identifying bioactive SP in this plant increases its potential use as source for developing anticoagulant drugs. Thus, in addition to this study, *E. crassipes* has been selected further for bioguided fractionation and isolation of active anticoagulant polysaccharides.

## 3. Experimental Section

### 3.1. Plant Material

The following plants were collected in the Agua Quente stream (05°59′ S and 35°07′ W), located in Parnamirim, Rio Grande do Norte state, Brazil: *Eichhornia crassipes* (Mart.) Solms (Liliopsida-Commelinales), *Egeria densa* Planch (Liliopsida-Alismatales), *Egeria naja* Planchon (Liliopsida-Alismatales), *Cabomba caroliniana* Gray (Magnoliopsida-Nymphaeales), *Ceratophyllum* sp. (Magnoliopsida-Ceratophyllales), *Hydrocotyle bonariensis* Comm. ex Lam. (Magnoliopsida-Apiales) and *Nymphaea ampla* (Salisb.) D.C. (Magnoliopsida-Nymphaeales). Specimens were identified by M. Iracema B. Loiola and deposited in the herbarium of the Center of Biosciences, Federal University of Rio Grande do Norte, Natal, Brazil, under catalog numbers 2260, 2259, 8107, 8105, 8106, 2258 and 2261 respectively.

The seaweeds *Caulerpa sertularioides*, *Gracilaria caudata*, and *Dictyopteris delicatula* were collected at Búzios Beach in Nísia Floresta-RN, Brazil. Algae were stored in our laboratory and dried at 50 °C with ventilation in an oven, ground in a blender and incubated with acetone to eliminate lipids and pigments.

### 3.2. Extraction of Polysaccharides

Plant samples were collected and transported to the laboratory in plastic bags containing river water. Plants were then cleaned and, when possible, each plant was divided into four parts: root, leaves, petiole and rhizome. Each part was dried at 50 °C with ventilation and ground in a blender. These materials were treated with acetone (4 L) in order to eliminate lipids and pigments. One hundred grams of each part, defatted, dry and powdered, were suspended with 500 mL of 0.25 M NaCl, and the pH was adjusted to 8.0 with NaOH. Twenty mg of maxatase, an alkaline protease from *Esporobacillus* (Biobras, MG, Brazil), was added to the mixture for proteolytic digestion. After 18 h of incubation at 60 °C under agitation, the mixture was filtered through cheesecloth. The filtrate was precipitated with 2 volumes of methanol and maintained at 4 °C during 24 h. The precipitate formed after centrifugation (10,000× g, 20 min.) was dried under vacuum and it was suspended in distilled water and then it was analyzed.

The sulfated polysaccharides from seaweed were obtained as described by Costa and colleagues [1].

### 3.3. Chemical Analysis

Total sugars were estimated using the phenol-H_2_SO_4_ reaction [25] with d-galactose as a base standard. After acid hydrolysis of polysaccharides (4 N HCl, 100 °C, 4 h), sulfate content was measured using the gelatin-barium method [26]. Polysaccharides were hydrolyzed with 0.5, 1, 2, and 4 M of HCl, respectively, for varying periods of time (0.5, 1, 2 and 4 h) at 100 °C. Reducing sugars were determined using the Somogyi-Nelson method [27]. Following acid hydrolysis, sugar composition was determined by a LaChrom Elite^®^ HPLC system from VWR-Hitachi with a refractive index detector (RI detector model L-2490). A LichroCART^®^ 250-4 column (250 mm × 40 mm) packed with Lichrospher^®^ 100 NH2 (5 μm) was coupled to the system. The sample mass used was 0.2 mg and analysis time was 25 min. The following sugars were analyzed as references: arabinose, fructose, fucose, galactose, glucose, glucosamine, mannose, rhamnose, and xylose. The amount of uronic acid was determined as described by Barroso *et al*. [28]. Protein content was measured using the methods described by Spector [29], with bovine albumin as standard.

### 3.4. Agarose Gel Electrophoresis

Agarose gel electrophoresis was prepared in 0.05 M 1,3-diaminopropane-acetate buffer, at pH 9.0 as previously described [17].

### 3.5. Fourier Transform Infrared Spectroscopy (FT-IR)

Sulfated polysaccharides (5 mg) were mixed thoroughly with dry potassium bromide. A pellet was prepared and the infrared spectra between 500 and 4000 cm^−1^ were measured on a Thermo-Nicolet Nexus 470 ESP FT-IR spectrometer. Thirty-two scans at a resolution of 4 cm^−1^ were averaged and referenced against air.

### 3.6. Histological Examination

Plant samples were collected in the same area described above, transported to the laboratory in plastic bags containing river water and cleaned of other organisms. Fragments 5 mm in diameter were cut from the roots and immediately fixed in 70% FAA mixture (formalin 40%, alcohol 70%, acetic acid) for 48 h, and then conserved in 70° G.L. ethyl alcohol. For anatomic analysis, these materials were dehydrated with acetone graded series followed by the addition of paraffin wax in accordance with standard techniques applied in plant anatomy. Sections (10 μm) were obtained in a microtome (Sorvall, Asheville, NC) and stained with 1% toluidine blue (Sigma), pH 4.4, for 3 min at 40 °C. This stain reveals the presence of sulfated polysaccharides by metachromasia [9].

### 3.7. Scanning Electron Microscopy (SEM) and Energy-Dispersive X-ray Analysis (EDXA)

Pieces of ~9 mm^2^ from the roots of *E. crassipes* were cut with razor blades under a stereomicroscope and rinsed with a 0.1 M cacodylic acid buffer, pH 7.4 and kept at room temperature for 60 min in 2.5% glutaraldehyde. After washing in the cacodylic acid buffer, roots were post-fixed and contrasted for 1 h in 1% osmium tetroxide (OsO4). Fixed roots were then dehydrated with increasing concentrations of ethanol, dried using the CO_2_ critical-point method, sputter-coated with gold and examined with a Shimadzu SSX-550 microscope.

Elemental composition of roots was determined by energy dispersive X-ray analysis (EDXA). Samples were prepared for MEV as described above, and analyzed on a Shimadzu SSX-550 equipped with a Noran-Voyager analytical system. A focused spot (*d* ~ 100 nm) was used to analyze areas of interest in the root sample. Areas probed were representative of the whole sample (we obtained several spectra from the individual particles). Micrographs were obtained from untilted samples (before analysis). During EDXA, the samples shown in this investigation were stable under the electron beam. Typical acquisition data were: accelerating voltage = 20 kV, live-time = 300 s, dead-time ~ 18%, and sample tilt angle = 30°.

Chemical mapping was performed as described by Andrade *et al*. [30] using a Shimadzu SSX-550 microscope.

### 3.8. Abiotic Factors

Water from the Água Quente stream was collected at three different sites and pH and salinity of each solution were determined using a PHTEK pHS-3B pH meter and an RTS 101 ATC refractometer, respectively.

### 3.9. Proximate Composition

Proteins, carbohydrate, lipids, ash, moisture and nitrogen contents of plants were determined by standard AOAC (1995) [31] methods. Protein content was calculated by converting the nitrogen content, determined by the micro-Kjeldahl method (6.25·N). Carbohydrate content was determined as the weight difference using protein, lipid, fiber, moisture and ash content data. Lipids from plant powder were extracted in a Soxhlet extractor (TECNAL-TE-044, São Paulo, BR) using hexane. The residue was re-extracted, after being washed with boiling distilled water and acetone and finally dried at 105 °C to constant weight. The material was heated at 550 °C for 3 h and the weight recorded. Moisture content was established by drying the plant samples in an oven at 105 °C until a constant weight was obtained. Ash content was obtained by calcinations in a muffle furnace at 550 °C for 4 h.

### 3.10. Genomic Analyses

BLAST analysis was performed at NCBI [32] as described for Altschul and colleagues [33]. Nucleotide and protein sequences from *Homo sapiens*, *Rattus norvegicus*, *Mus musculus*, *Cricetulus longicaudatus*, *Drosophila melanogaster*, *Mus* sp., *Flaveria bidentis*, *Rhizobium* sp. were used as probes for this search. Homologous sequences were then researched in plant databases at NCBI GenBank [32], TAIR [34], TIGR [35]. Additionally, sequences M92042; U17970 and U02304 were used to search for 5′PSB and 3′PB in the conserved protein region for sulphotransferase. Primers were designed using these motifs. *E. crassipes* DNA was extracted as described for Doyle and Doyle [36]. PCR reactions were performed using 100 ng of DNA and PCR conditions were as follows: 3 min at 95 °C, then 35 cycles of 95 °C 30 s, 50–62 °C 30 s (non-stringent or stringent temperature condition), 72 °C 30 s, and then 72 °C for 5 min. Taq polymerase (Invitrogen) was used in these reactions, and the primers were: P1F 5′-TCT ACC TAT GAG CCA GTG CTG-3′, P1R 5′-CAG AGG TGG TGT TGG AGG GAA TGA-3′, P2R 5′-CTG AGC CAG CGT TCA ATA TGA GTG-3′, PF 5′-AGG AGA AGA CAT GTG ATC GCT TCC-3′ and PR 5′-CCA GCG TTC AAT ATG AGT GGC ATA-3′. The amplified fragments were separated on a 0.8% agarose gel.

### 3.11. Anticoagulant Activity

All protrombin time (PT) and activated partial thromboplastin time (APTT) coagulation assays were performed with a coagulometer as previously described [37], and measured using citrate treated normal human plasma. All assays were performed in duplicate and repeated at least three times on different days (*n* = 6).

## 4. Conclusions

In conclusion, we found sulfate in polysaccharide-rich extracts of three freshwater plants: *E. crassipes*, *N. ampla* and *H. bonariensis.* All parts of *E. crassipes* synthesize SP; however, significant amounts were only recorded in the roots. We extracted SP from *E. crassipes*, which showed anticoagulant activity at different levels. It is evident that the biological function of SP is dependent on its structural characteristics. Additional investigation will identify and purify active polysaccharides in *E. crassipes* to further our understanding of the complete polysaccharide structure, including the composition and sequence of monosaccharides, the configuration and position of glycosidic linkages, the position of branching points and the structure-function relationship. This will certainly provide a promising opportunity to elucidate the biological roles of polysaccharides and develop potential anticoagulant drugs based on the three-dimensional structures.

## Figures and Tables

**Table 1:** Mass/mass ratio of total sugars, sulfate and proteins extracted from different portions of plants.

Plant	Portion	Polysaccharides	Sulfate	Protein
*Eichhornia crassipes* (Mart.) Solms.	Root	1.00	0.240	0.15
	Rhizome	1.00	0.140	0.11
	Petiole	1.00	0.340	0.20
	Leave	1.00	0.060	0.19

*Egeria densa* Planchon	Root	1.00	-	0.04
	Petiole	1.00	-	0.02
	Leave	1.00	-	0.02

*Egeria naja* Planchon	Stem	1.00	-	0.05
	Leave	1.00	-	0.06

*Cabomba caroliniana* Gray	Stem	1.00	-	0.05
	Leave	1.00	-	0.06

*Ceratophyllum aquaticum* Lam.	Root	1.00	-	0.01
	Stem	1.00	-	0.08
	Leave	1.00	-	0.11

*Hydrocotyle bonariensis* Comm. ex Lam.	Root	1.00	-	0.03
	Petiole	1.00	0.010	0.04
	Leave	1.00	0.290	0.06

*Nymphaea ampla* (Salisb.) D.C.	Root	1.00	0.240	0.06
	Rhizome	1.00	0.040	0.13
	Petiole	1.00	0.003	0.11
	Leave	1.00	0.008	0.01
	Flower	1.00	0.020	0.04

**Table 2:** Proximate composition of root, rhizome, petiole, and leaf of *E. crassipes*.

Compound	Root	Rhizome	Petiole	Leaf
Carbohydrate (%)	13.6	13.7	13.6	11.9
Ash (%)	14.6	13.6	6.6	5.9
Protein (%)	9.1	8.9	13.7	13.9
Lipids (%)	1.1	1.6	3.0	1.5
Moisture (%)	91.3	93.2	93.2	86.5
Nitrogen (%)	1.5	1.4	2.2	2.2

**Table 3:** Monosaccharide composition of sulfated polysaccharides extracted from *E. crassipes*.

Portion			Molar	Ratio	

	Gal 1	Glc 1	Ara 1	Xyl 1	Man 1
Root	1.0	0.5	0.3	0.1	0.3
Rhizome	1.0	0.4	0.7	0.1	0.1
Petiole	1.0	0.5	0.6	-	-
Leaf	1.0	1.0	1.0	-	-

1 Molar ratio of sugars using galactose as parameter. *Gal*, galactose; *Glc*, glucose; *Ara*, arabinose; *Xyl*, xylose; *Man*, mannose.

**Table 4:** Major components elemental surface composition of root the *E. crassipes* determined by SEM/EDXA.

Main components (at%)			

Elements	Epidermis	Cortex	Vascular bundle
C	30.1	19.1	10.5
O	37.3	52.4	49.3
N	29.8	28.4	38.7
S	2.5	0.9	1.3

**Table 5:** Anticoagulant activity of sulfated polysaccharides from different sources.

Sample	Sulfate/sugar (w/w)	a PT	a APTT
SP from Root	0.24	nd	0.100
SP from Rhizome	0.14	nd	0.150
SP from Petiole	0.34	nd	nd
SP from Leaf	0.06	nd	0.100
SP from *C. sertularioides*	0.85	nd	0.100
SP from *G. caudata*	0.20	nd	nd
SP from *D. delicatula*	0.34	nd	0.040
Heparin	-	0.001	0.002

SP: sulfated polysaccharides; APTT: activated partial thromboplastin time; PT: protrombin time; nd: anticoagulant activity not detected at concentrations tested (from 0.001 to 0.2 mg/mL); - not determined;

a Data are reported as concentration (mg/mL) required to double APTT compared to saline control.
